# XenofilteR: computational deconvolution of mouse and human reads in tumor xenograft sequence data

**DOI:** 10.1186/s12859-018-2353-5

**Published:** 2018-10-04

**Authors:** Roelof J. C. Kluin, Kristel Kemper, Thomas Kuilman, Julian R. de Ruiter, Vivek Iyer, Josep V. Forment, Paulien Cornelissen-Steijger, Iris de Rink, Petra ter Brugge, Ji-Ying Song, Sjoerd Klarenbeek, Ultan McDermott, Jos Jonkers, Arno Velds, David J. Adams, Daniel S. Peeper, Oscar Krijgsman

**Affiliations:** 1 Central Genomic Facility, Netherlands Cancer Institute, Amsterdam, The Netherlands; 2 Division of Molecular Oncology and Immunology, Netherlands Cancer Institute, Plesmanlaan 121, 1066 CX Amsterdam, The Netherlands; 3 Division of Molecular Pathology, Netherlands Cancer Institute, Amsterdam, The Netherlands; 4 Division of Molecular Carcinogenesis, Netherlands Cancer Institute, Amsterdam, The Netherlands; 5 Experimental Cancer Genetics, Wellcome Trust Sanger Institute, Hinxton, Cambridgeshire, UK; 6 The Wellcome Trust/Cancer Research UK (CRUK) Gurdon Institute, University of Cambridge, Cambridge, UK; 7 Division of Experimental Animal Pathology, Netherlands Cancer Institute, Amsterdam, The Netherlands; 8 Cancer Genome Project, The Wellcome Trust Sanger Institute, Hinxton, Cambridge, CB10 1SA UK; 9 Present address: DNA Damage Response Biology, Bioscience Oncology IMED Biotech Unit, AstraZeneca, Cambridge, CB4 0WG UK

**Keywords:** Sequencing, Xenograft, Cancer, Next-generation sequencing (NGS), Melanoma, Breast cancer, Patient-derived xenografts (PDX)

## Abstract

**Background:**

Mouse xenografts from (patient-derived) tumors (PDX) or tumor cell lines are widely used as models to study various biological and preclinical aspects of cancer. However, analyses of their RNA and DNA profiles are challenging, because they comprise reads not only from the grafted human cancer but also from the murine host. The reads of murine origin result in false positives in mutation analysis of DNA samples and obscure gene expression levels when sequencing RNA. However, currently available algorithms are limited and improvements in accuracy and ease of use are necessary.

**Results:**

We developed the R-package XenofilteR, which separates mouse from human sequence reads based on the edit-distance between a sequence read and reference genome. To assess the accuracy of XenofilteR, we generated sequence data by in silico mixing of mouse and human DNA sequence data. These analyses revealed that XenofilteR removes > 99.9% of sequence reads of mouse origin while retaining human sequences. This allowed for mutation analysis of xenograft samples with accurate variant allele frequencies, and retrieved all non-synonymous somatic tumor mutations.

**Conclusions:**

XenofilteR accurately dissects RNA and DNA sequences from mouse and human origin, thereby outperforming currently available tools. XenofilteR is open source and available at https://github.com/PeeperLab/XenofilteR.

**Electronic supplementary material:**

The online version of this article (10.1186/s12859-018-2353-5) contains supplementary material, which is available to authorized users.

## Background

Cancer research heavily relies on model systems such as cell lines. These cell lines have typically been cultured for decades and only partially recapitulate the genetic features of patient tumors [1]. More advanced clinical cancer models are the cell line-derived xenograft and patient-derived xenografts (PDX) [2]. With this approach, either a cancer cell line or a patient tumor sample is injected or transplanted into a host, generally immunodeficient mice. In these xenografts, the complex interactions between the tumor and its microenvironment are at least partially recapitulated, as is the heterogeneity in tumors in the case of PDX [3–8]. For these reasons, xenograft models might serve as a better proxy for human tumor samples and have become indispensable for development, validation and optimization of cancer treatment regimens [1, 2, 9]. Despite its limitations [8, 10], the wide applicability of PDX, and more generally of tumor xenografts, is reflected by tens of thousands publications describing numerous biological, mechanistic and preclinical applications [11–16].

In spite of this tremendous popularity, sequence analysis of RNA or DNA from tumor xenograft and PDX samples is challenging: the sequence data contain not only DNA and RNA from the grafted human tumor cells but also from the mouse, mostly due to infiltrating stromal cells [17]. When sequencing the combined ‘bulk tumor’ DNA, sequence reads originating from the mouse result in false positive single nucleotide variants (SNV) when calling mutations [18]. Similar challenges are observed when sequencing RNA: beside false positive SNVs, the gene expression levels are often obscured by reads that derive from mouse cells [19]. Despite mouse-derived sequence reads representing a potential source of bias in sequence analysis of tumor xenografts, the number of tools to solve this important issue is surprisingly limited.

Some solutions have been proposed to bioinformatically remove mouse host sequences from the analysis. The most straightforward method is to map all reads first to the mouse reference genome. Sequence reads failing to map are remapped to the human reference, which is followed by standard downstream analyses [20]. A major disadvantage of this method is that human reads from evolutionary conserved regions will also map to the mouse reference genome. Such reads are inadvertently removed from further analysis, which erodes the read depth and thus sensitivity of variant detection in DNA sequencing. Similarly, it erodes gene expression estimates (counts) when sequencing RNA.

An improved version of this concept, developed for RNA sequence data but also applicable to DNA sequence data, uses a so-called k-mer approach with a mixed mouse/human reference set [19]. This method catalogs for every possible sequence of length *k*, its presence in the human and mouse reference genome sequences. If a k-mer is unique to one reference, its occurrence in sequencing data indicates the species’ origin. Distinction between conserved regions, which are also the most problematic in cross strain filtering, would require long k-mers. However, k-mer elongation rapidly increases computer memory requirements and is therefore less feasible.

Deconvolution based on the alignments of sequence reads to a human and mouse reference genome separately has also been proposed [21, 22]. This method utilizes the alignment scores of each sequence read to the mouse and human reference genome to categorize reads as human or mouse. Both methods shows a much better performance as compared to filtering for reads that do not map to the mouse reference genome [19]. However, the number of supported, open-source solutions are limited and improvements in accuracy and ease of use are necessary.

The challenges in the analysis of sequence data from xenografts and the limited availability of tools motivated us to firstly provide a detailed study into the effect of mouse reads on subsequent analyses. Furthermore, we set out to develop a method for accurate filtering on species’ origin using a procedure that is easily applicable in bio-informatics pipelines to improve analysis of DNA and RNA sequence data from xenografts.

### Implementation

**Fig. 1:**
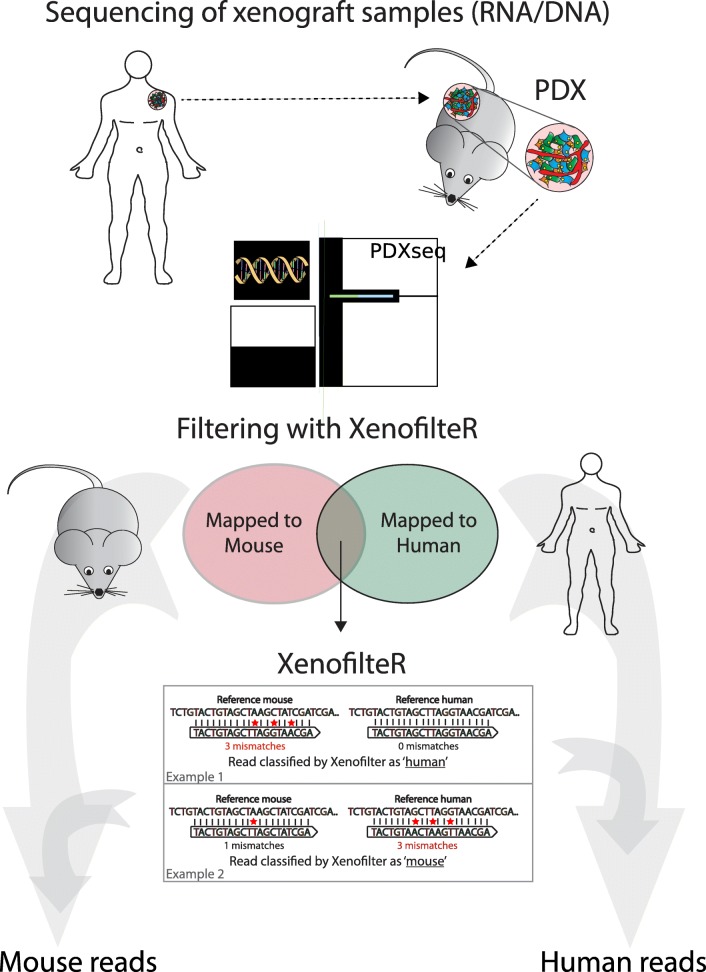
Overview of XenofilteR workflow. Sequence reads (fastq) from PDX are mapped with the appropriate aligner (e.g. BWA, Tophat, STAR) to both a human and mouse reference genome. Sequence reads that only map to a single reference genome are classified to that specific organism. For seqeunce reads that map to both the human and mouse reference genome the edit distance is calculated which is defined by the number of base pairs different between the sequence read and the reference genome. Next, XenofilteR classifies the sequence reads as ‘human’ or ‘mouse’ based on the edit distance

XenofilteR is an easy-to-use R-package for deconvolution of mouse and human sequence reads form xenograft sequence data. XenofilteR takes a file with 2 bam files (e.g. BWA [23], TopHat [24], STAR [25]) for each sample as input: reads aligned to the human reference and reads aligned to the mouse reference genome (Fig. 1). XenofilteR does not require a specific order of the sequence reads for the input BAM files. Default output of XenofilteR is a new bam file with the sequence reads classified as human. Optionally, a second bam file can be generated with the sequence reads classified as mouse.

### Filtering

Sequence reads that only map to a single reference genome are classified to that specific organism. For reads that map to both the human and mouse reference genome the edit distance is calculated by summing soft clips, insertions (both derived from the CIGAR string) and the number of mismatches (bam tag: ‘NM’) (Fig. 1). For paired-end sequencing, the edit distance of the forward and reverse read is averaged. Sequence reads with an equal edit distance to mouse as well as human are not in either bam file as these cannot be assigned. Assignment of reads (or read pairs) to either human or mouse is based on the edit distance, with reads having a lower edit distance for the reference genome of a species being classified as originating from that species.

Although sequence reads generally map to one specific location on the genome, some reads can be mapped reasonably well to multiple places on the genome, these mappings are called secondary alignments. In XenofilteR, the edit distance is calculated on the primary alignments only. All secondary alignments are either kept in the filtered output or removed depending on the classification based on the primary alignment. Classification can further be fine-tuned by setting a maximum number for the edit distance (default = 4) and a penalty for unmapped reads in case of paired-end sequencing (default = 8).

### Parallel implementation and computational time

XenofilteR uses functionality from *GenomicAlignments* and *Rsamtools* [26] for reading and manipulating bam files. Parallel analysis is implemented in XenofilteR package using *BiocParallel*. As XenofilteR only evaluates the sequences that map to both reference genomes and requires only little information from the bam files, we were able to minimize the CPU time and memory needed for analysis. XenofilteR can be run on a desktop computer in single sample mode and in parallel on computer servers. Examples of code to run XenofilteR and further documentation is available at (https://github.com/PeeperLab/XenofilteR).

## Results

### Mouse sequence reads map to specific regions on the human genome

**Fig. 2:**
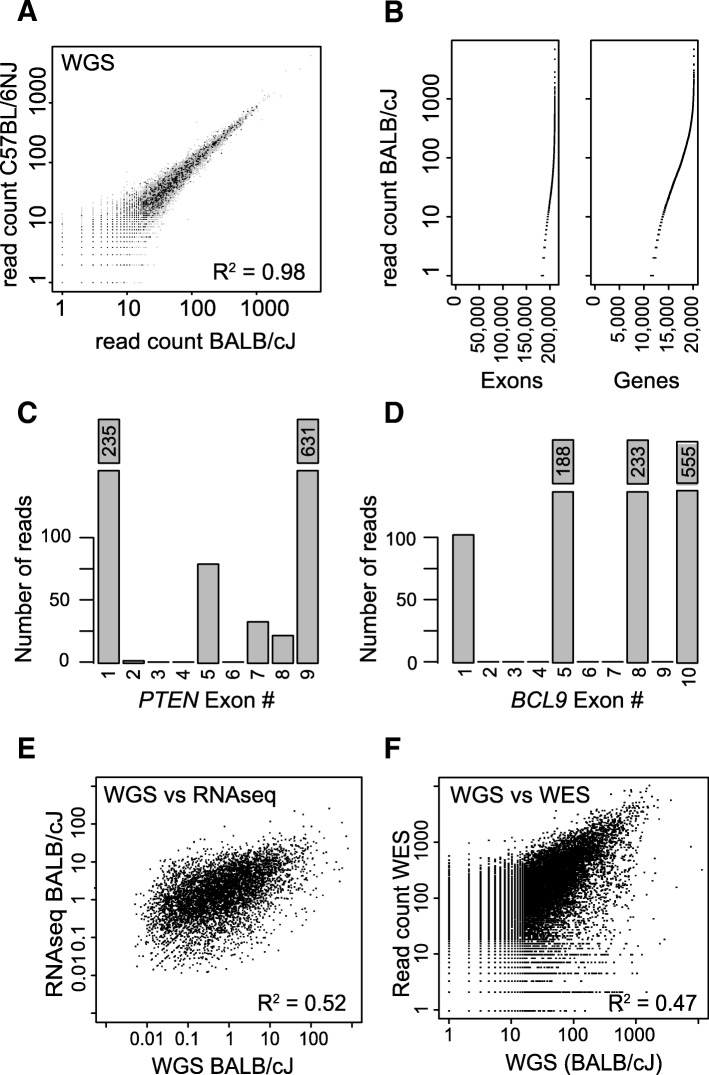
Mapping of mouse DNA and RNA to the human reference genome. **a**: Pair-wise comparison of the number of sequence reads per exon from mouse WGS (BALB_Cj versus C57BL_6NJ) mapped to a human reference. **b**: Number of reads (log10) that originate from mouse that mapped to the human reference, sorted by reads count; per exon (left panel) and per gene (right). **c**: Number of mouse reads from WGS that mapped to the human gene *PTEN*. **d** Number of mouse reads from WGS that mapped to the human gene *BCL9*. **e**: Comparison for read count of BALB_Cj RNAseq and WGS, both mapped to a human reference. Read count is corrected for exon length. **f**: Comparison for exon read count of WGS and WES of mouse DNA, both mapped to a human reference. WES on mouse DNA was performed with a human-specific enrichment kit

In xenograft models, human tumors are grown in a murine host. Sequence data of these tumor xenografts commonly contain reads that originate from the host. To investigate which genes and exons are likely to be affected by mouse reads, we mapped whole genome DNA sequence data (WGS) of three mouse strains (NOD/ShiLtJ, BALB/cJ and C57BL/6NJ) [27, 28] to a human reference [29]. On average, 0.3% of mouse reads mapped to the human reference genome, of which 18–20% overlapped with an exon of a protein-coding gene. A high correlation was observed in the number of reads mapped to exons between different mouse strains (R^2^ = 0.98, Fig. 2a). Mouse reads mapped to specific regions of the genome with ~ 2000 (out of 200.000 exons in total) exons exceeding 100 reads, including exons from known cancer driver genes [30] (Fig. 2b, Additional file 1: Table S1). Mapping of BALB/cJ WGS data to the human reference revealed that 13% of exons have at least a single mouse read mapped, affecting 43% of genes in total (Fig. 2b). For example, out of the ten exons of *BCL9*, four exons had over 100 mapped reads mapped, the remaining six had only a few reads or none at all (Fig. 2d). Similar results were observed for other cancer-related genes such as *PTEN* (Fig. 2c).

Also, RNA sequence data of the same three mouse strains (NOD/ShiLtJ, BALB/cJ, C57BL/6NJ) [27, 28] were mapped to the human reference genome. As the sequence similarity between mouse and human is highest for the coding regions, the number of RNA sequencing reads that map to the human reference is much higher (4–8% of reads) compared to WGS. The read count per gene from the RNA sequence data correlated (R^2^ = 0.52) with read count per gene in the WGS (Fig. 2e), indicating that the same exonic regions are affected with WGS and RNAseq.

Although mouse RNA sequencing and WGS data clearly showed that mouse reads can map to the human reference genome, both methods were performed on the complete RNA and DNA pools of the sample. Whole exome sequencing (WES) on the other hand, includes an enrichment step using baits designed to target exons on the human reference genome. To test the affinity of mouse sequence reads to the human baits, we sequenced eight mouse DNA samples enriched with a human exome kit (Illumina, SureSelect Human Exon Kit 50 Mb capture set, Agilent, G3362). On average, 29.2 million reads were sequenced per sample of which ~ 11% could be mapped to the human reference genome. Furthermore, 85–86% of mapped reads did so to an exon. These findings were highly reproducible, with a high correlation in exon read count between samples (R^2^ = 0.93–0.98) but also with the results from WGS (BALB/cJ, R^2^ = ~ 0.47), albeit a higher average read count was observed per exon with WES (Fig. 2f). To summarize, mouse sequence reads map to specific regions on the human genome, an issue that we have observed for RNA sequencing, WGS and WES.

### Sequence reads of mouse origin affect downstream analysis of xenografts

**Fig. 3:**
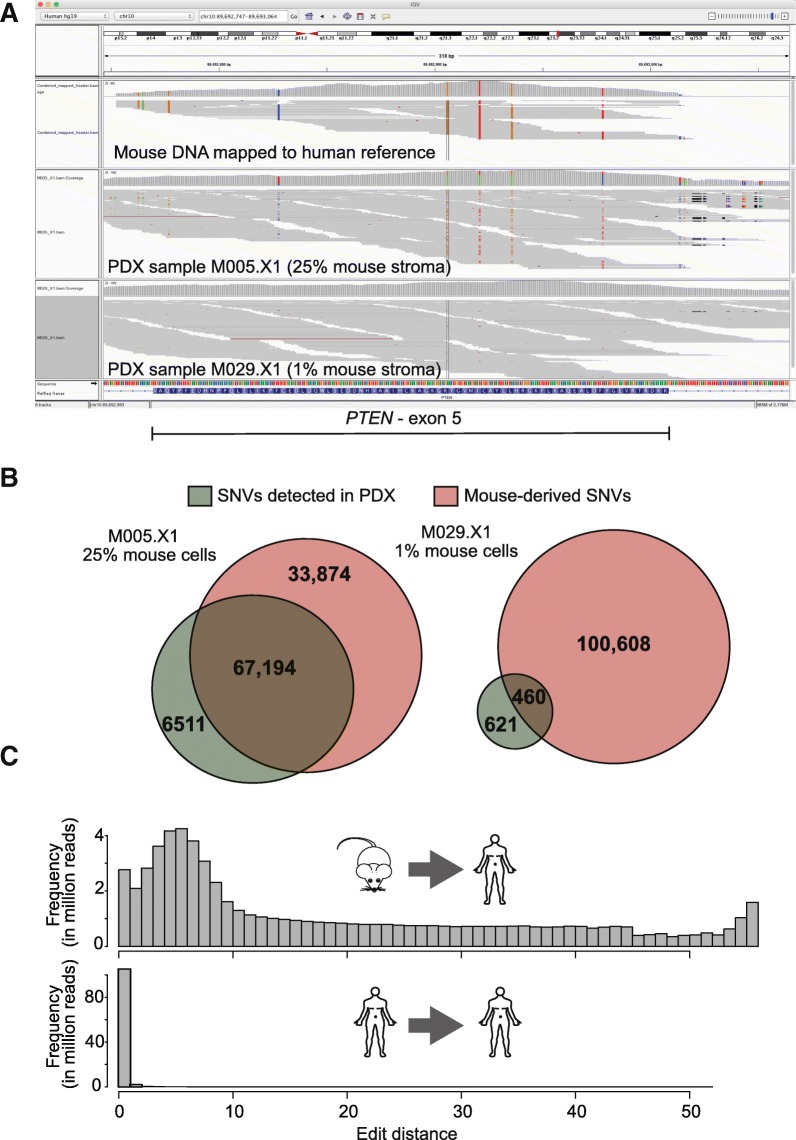
The effect of mouse reads in PDX samples. **a**: Integrative Genome Viewer (IGV) image of exon 5 of *PTEN.* Top panel shows mouse DNA mapped to the human reference genome, middle panel melanoma PDX sample M005.X1 with 25% mouse stroma and bottom panel melanoma PDX sample M029.X1 with 1% of mouse stroma. Each grey horizontal line represents a single sequence read. Base pair differences between human reference genome and sequence reads (SNV) are indicated with a color (depending on the base pair change). **b**: Overlap between somatic SNVs detected in PDX, with high percentage mouse stroma (M005.X1), and low percentage of mouse stroma (M029.X1). **c**. The edit distance of sequence reads from mouse DNA aligned to a human reference genome (top panel) and from human DNA mapped to a human reference genome (bottom panel)

In recognition that mouse reads can map to the human reference genome, we set out to determine the effect that these reads have on analyses of eight PDX samples [14]. For each sample, the percentage of mouse stroma was estimated by two pathologists and averaged (Additional file 2: Table S2). Mutation analysis on the WES data of the PDX samples revealed an extremely high number of single nucleotide variants (SNVs), especially in the samples with a high percentage of mouse stroma. A direct comparison of PDX samples containing a high number of mouse sequence reads, mapped to the human reference, revealed that many of the SNVs in the samples overlap with SNV that originate from mouse, for example in one of the exons of *PTEN* (Fig. 3a).

Genome-wide mutation analysis on the mouse WGS data mapped to the human reference identified 101,068 SNVs (19.5% exonic). Intersection of this list with the lists of SNVs detected in the PDX samples suggested that many SNVs detected in PDX samples are derived from reads that originate from mouse cells. In the PDX sample M005.X1 (~ 25% mouse stroma), 73,705 SNVs were detected, of which 67,194 overlapped with the 101,068 SNVs from mouse reads mapped to the human reference. The PDX sample M029.X1 (~ 1% mouse stroma) had a much lower total number of SNVs, only 460 detected SNVs in the PDX samples overlap with the mouse SNVs (Fig. 3b). In conclusion, sequence reads that originate from mouse have a large effect on mutation calling on samples derived from PDX.

### The edit distance can be used to classify sequence reads

Accurate assignment of reads to either mouse or human is pivotal to assure high quality downstream analyses. Currently available tools generally use the mapping of reads to a combined reference genome or to both genomes as a classification strategy [18, 19]. However, due to the sequence similarity between mouse and human, the mapping itself might not provide the optimal distinction between reads of human and mouse origin.

A striking distinction between the alignments to mouse and human reference was the difference in ‘edit distance’: the number of base pairs in a given mapped read that discord with the reference genome. To illustrate this difference, we used two samples, a WES of a patient melanoma sample (M032) [8] and a WES of mouse DNA enriched in silico with human baits to mimic PDX samples. Both samples were mapped to the human reference genome. Only 4% of mouse DNA reads showed an edit distance of 1 or lower, as opposed to 96% of human DNA reads (Fig. 3c). Thus, the edit distance of a sequence read can be used to filter mouse from human sequence reads.

Based on these observations, we developed an algorithm, called XenofilteR, which calculates the edit distance for each read that maps to both the human and mouse reference genomes (Fig. 1). The edit distance is calculated by summing soft clips, insertions (both derived from the CIGAR string) and the number of mismatches (bam tag: ‘NM’). The reference genome to which a specific sequence read has the lowest edit distance is considered as the species of origin for that read. By differentiating each sequence read in the original input bam files, XenofilteR generates a new bam file, which contains the sequence reads classified as human only. Conversely, XenofilteR can also output the bam file with all reads classified as mouse. XenofilteR is programmed in R and publically available from GitHub (https://github.com/PeeperLab/XenofilteR).

### XenofilteR accurately filters mouse reads from human reads from in silico-mixed datasets

**Fig. 4:**
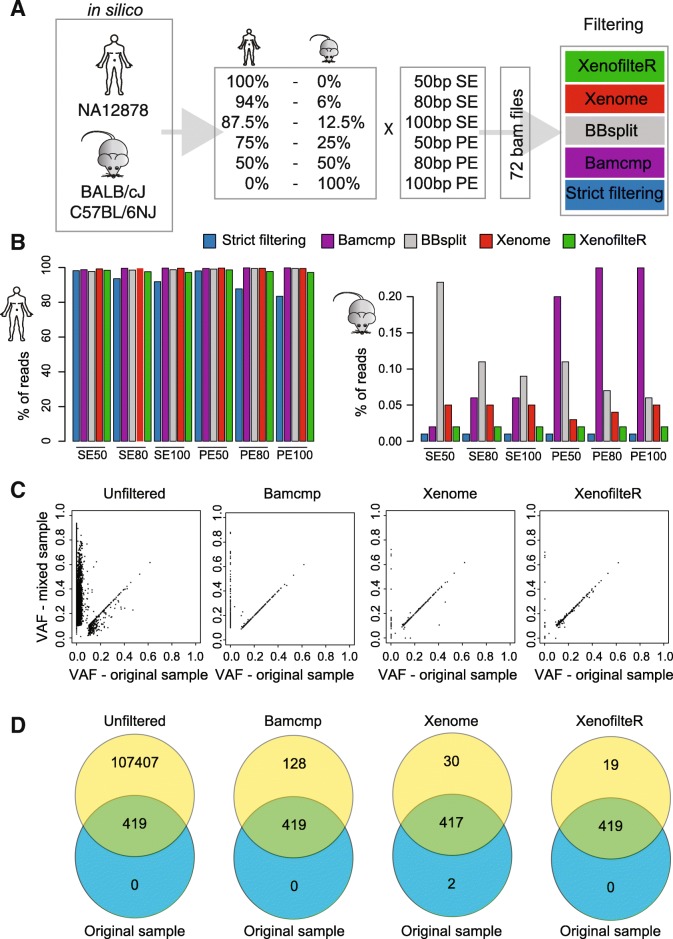
Performance of strict filtering, bamcmp, Xenome and XenofilteR on in silico mixed samples. **a**. Schematic overview of samples, dilutions and sequence read type for generation of the samples mixed in silico. **b**. Percentages of sequence reads remaining per species after filtering with strict filtering, bamcmp, Xenome and XenofilteR options for the 50:50, mouse (BALB/Cj):human (NA12878) WGS mixes. **c**. Variant Allele Frequency (VAF) of the SNVs in the original sample compared to unfiltered and filtered samples after in silico-mixing with mouse sequence reads. **d**. Venn diagrams of non-synonymous mutations in the original sample with filtered and unfiltered samples

To validate this computational method and compare the results to other available methods, we generated fastq files containing both mouse [27, 28] and human [29] WGS reads. We generated paired-end and single-end fastq files of different sequence length and multiple percentages of mouse cells (Fig. 4a and Additional file 3: Table S3). These files were generated for two mouse strains (BALB/cJ, C57BL/6NJ; a full description on how the files were generated is available in the methods section). The combined fastq files were mapped to both human and mouse references (C57BL/6NJ). We applied five tools to the generated data: XenofilteR Strict filtering (filtering of all reads that map to mouse), bamcmp [21], BBsplit [22], Xenome [19] and XenofilteR (all with default settings). Since the origin of each read was known, we could calculate the accuracy of each of the three methods. Because the C57BL/6NJ mouse strain is identical to the mm10 reference genome the most accurate classification was reached with this mouse strain compared to BALB/cJ (Additional file 3: Table S3).

Results from the dataset with mixed human and BALB/cJ reads strain shows that for all tools true and false positive classification of reads as human depend on both sequence length and on whether sequencing was paired-end, but not on the initial percentage of mouse reads in the mixture (Fig. 4b and Additional file 3: Table S3). Although the Strict filtering method showed the least misclassified mouse reads (0.01%), it was accompanied by a severe decrease in the number of correctly assigned human reads (Fig. 4b). By contrast, both XenofilteR and Xenome correctly identified almost all mouse reads with, respectively, less than 0.02 and 0.04% of mouse sequence reads remaining after filtering. Bamcmp retained the highest number of human reads but also kept a high percentage of mouse sequence reads, especially for the paired-end sequence runs (> 0.20%). Similar results were observed for BBsplit, except that a high number of mouse sequence reads were kept both with single-end and paired-end sequencing (Fig. 4b and Additional file 3: Table S3).

In addition to the WGS of in silico mixed samples, we also determined the effect of filtering on the detection of somatic variants in a cancer sample. For this purpose, we mixed in silico WES sequence reads of a patient tumor sample M032 [8] with those obtained from mouse WES, with both sequence libraries generated using the same human exome enrichment kit, in a 3:1 ratio. This sample was processed in parallel with Bamcmp, Xenome and XenofilteR. Due to the high number of erroneously filtered sequence reads the performance of both the Strict Filtering method and BBsplit was not further investigated. All three methods were run with default settings followed by mutation calling (GATK). In the original tumor sample, 419 somatic SNVs were detected; in the mixed sample, without exclusion of mouse reads, a total of 107,826 SNVs were observed, comparable to the number of SNVs in PDX sample M005.X1. Filtering with Bamcmp, Xenome or XenofilteR resulted in 547, 449 and 438 SNVs, respectively. The 438 SNVs remaining after XenofilteR filtering included all 419 SNVs identified in the original samples, with almost identical VAFs (Fig. 4c), and an additional 15 false positive SNVs (Fig. 4d). This is an improvement over Bamcmp and Xenome, which both produced more false positives, 128 and 30 respectively (Fig. 4d). In addition, for two SNVs, the VAF was lower after filtering compared to the original tumor (Fig. 4c). Thus, when filtering samples with in silico-mixed mouse and human sequence reads, XenofilteR improves on Bamcmp and Xenome both regarding total number of filtered sequence reads and in retaining mutations of human origin.

### XenofilteR accurately filters mouse reads from human reads in PDX samples

**Fig. 5:**
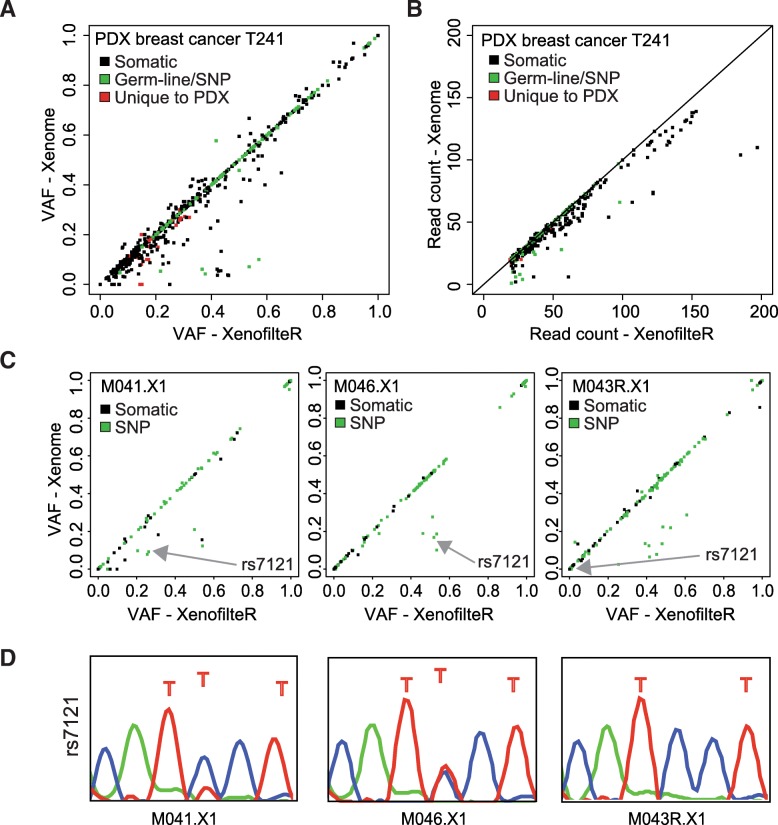
Performance of XenofilteR and Xenome on PDX samples. **a**: Mutation calling on exome sequence data of a breast cancer PDX sample. The variant allele frequency (VAF) was plotted after filtering with XenofilteR (x-axis) and Xenome (y-axis). Plotted in black are mutations also detected in the patient sample, in green known SNPs and in red SNVs detected in the PDX only. **b**: Read count of each SNV used to calculate the VAF from A for Xenome and XenofilteR. **c**: Mutation calling on targeted sequencing of melanoma samples. In green all known SNPs are indicated, in black the remaining SNVs. **d**: Validation of the SNP rs7121 (*GNAS*) by Sanger sequencing with human-specific primers

In addition to in silico-mixed samples, we tested XenofilteR on PDX samples and compared the results to those obtained with the best performing method on the in silico data, Xenome. Patient tumor, normal and PDX were analyzed by WES for three breast cancer samples. Mutations were called on these samples after XenofilteR or Xenome filtering (Fig. 5a). For each SNV identified in the filtered PDX, we traced whether it was either also found in the patient tumor (Fig. 5a; black), in the matched normal or SNP database (Fig. 5a; green) or not found in either blood, SNP database or tumor sample (Fig. 5a; red). This last group represents either false positives or a difference between PDX and tumor (e.g. due to tumor heterogeneity or alternate sequence depth between patient tumor and PDX). However, similar to mutation calling in the in silico-mixed sample, the VAF was much lower for several mutations identified with Xenome compared to XenofilteR. This was reflected not only by the VAF but also by the read counts, on which the VAF was based (Fig. 4b): they were fewer after filtering with Xenome compared to XenofilteR in almost all cases. This suggests that Xenome might filter too stringently, which results in multiple SNPs and SNVs in the patient tumor to receive a VAF estimate below the true value.

In addition to the three PDX breast samples, we tested ten melanoma PDX samples for which targeted sequencing (using a 360-cancer gene panel) was performed [14]. We again calculated the VAF and indicated known SNPs (Fig. 5c and Additional file 4: Figure S1). Since only PDX were sequenced, no estimate exists for true somatic or germline mutations. Strikingly, and similar to the breast cancer analysis, the VAF of multiple SNVs and SNPs were lower after filtering with Xenome, compared to XenofilteR. Again, this suggests that XenofilteR filters are more sensitive, which contributes to its performance.

To further validate these findings, we selected two SNPs with discordant VAFs between XenofilteR and Xenome after filtering. We developed human-specific primers to perform Sanger sequencing on both SNPs. SNP rs7121, located in the gene *GNAS*, harbored a C > T change, in M041.X1and M046.X1, but not in M043R.X1, in concordance with the WES data. Also, the expected VAF of 50% was observed in the Sanger sequencing in M046.X1 and the VAF of ~ 25% was reflected in the lower peak for T in M041.X1 (Fig. 5d). SNP rs2071313, located in the gene *MEN1*, showed a G > T change in M041.X1 and M046.X1. Sanger sequencing revealed the SNP in M041.X1 as heterogeneous corresponding to the VAF after filtering with XenofilteR (Additional file 5: Figure S2A). In addition to the lower VAF, the number of sequence reads was much lower after filtering with Xenome, indicative of XenofilteR better representing the real VAF (Additional file 5: Figure S2B). Altogether, we conclude that XenofilteR outperforms Xenome for the analysis of mutation data of mixed human/mouse origin as illustrated by both in silico mixed data and subsequent corroboration in PDX samples from breast cancer and melanoma patients.

### XenofilteR allows for filtering of RNA sequencing data

The effect of mouse sequence reads on downstream analysis of PDX samples is not limited to DNA sequencing but affects RNA sequencing also. The method used by XenofilteR, for which classification is based on the edit distance of a read, can also be applied to RNA sequencing data, as the same values to calculate the edit distance are available in the BAM files (CIGAR and the tag NM). To validate whether indeed, filtering of RNA sequence PDX data can be accurately done, we applied XenofilteR on a set of seven PDX samples for which matched patient samples were available [14].

**Fig. 6:**
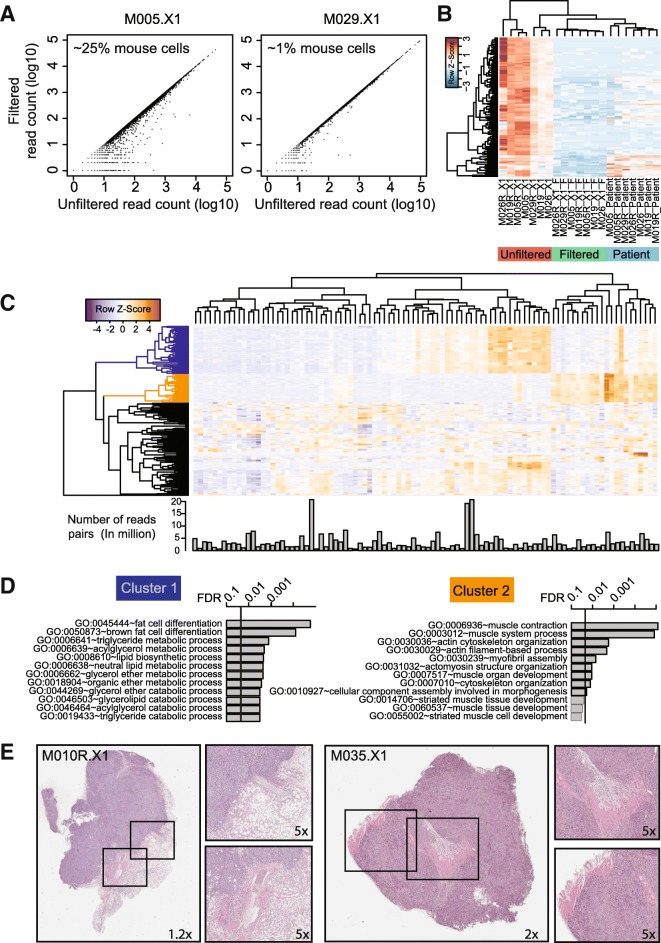
XenofilteR on RNAseq data. **a**. Scatter plots showing the number of RNA sequence reads before and after filtering for mouse sequence reads with XenofilteR both for a sample with a high percentage of mouse stromal cells (M005.X1) and a sample with a low percentage of mouse stromal cells (M029.X1). **b**. Cluster analysis of PDX samples before and after XenofilteR with matched patient samples. **c**. Heat map of the top 250 most variable mouse genes retrieved from a dataset of 95 PDX samples. Bar graph below the heat map shows the number of mouse sequence reads. **d**. Gene Ontology (GO) analysis of the top 2 clusters from **c**. **e**. H&E staining of a PDX sample with adjacent mouse fat cells (left) and a PDX sample with mouse muscle cells (right)

The effect of XenofilteR on the read counts in RNA sequence data was tested using two different samples, one with a high percentage of mouse cells (M005.X1, pathologist estimate was 25% of mouse cells) and one with a low percentage of mouse cells (M019.X1, 1% mouse cells). As expected, the largest difference between filtered and unfiltered read count was observed for sample M005.X1 (Fig. 6a).

Next, we compared the top differentially changed genes (FDR < 0.001) between filtered and unfiltered samples and generated a heat map and cluster analysis including the original patient samples (Fig. 6b). As expected, samples with the highest percentage of mouse cells also showed the highest expression of the selected genes. Most importantly, after filtering with XenofilteR the expression of the selected genes better reflected the expression of the genes in the patient samples.

We also tested XenofilteR on a large data set of 95 melanoma PDX RNA profiles. Although XenofilteR was initially developed to remove infiltrating mouse reads from PDX samples, we investigated whether the we could also use the method to select for mouse reads. For this purpose, XenofilteR was run on this large PDX cohort to remove the reads of human origin, leaving the mouse reads. As expected, considerable variation was observed with regards to the number of sequence reads classified as mouse, with a range from 408,145 to 20,725,475 sequence reads, with on average 6.1% of the total sequence reads classified as mouse (range: 1–35%). Cluster analysis based on the mouse read counts of the top 250 most variable genes showed separation in three clusters with clear expression patterns in specific samples for clusters 1 and 2 (Fig. 6c). Gene Ontology (GO) analysis of cluster 1 (blue) showed that this cluster was highly enriched for genes involved in fat cells and metabolic processes, suggesting the presence of mouse fat cells in this sample (Fig. 6c). We performed the same analysis for the genes in cluster 2 (orange) and found clear enrichment for genes related to muscle cells (Fig. 6c). Both cell types likely represent the predominant components of the murine microenvironment associated with subcutaneous tumor xenografts. Indeed, pathological examination of the H&E stainings confirmed that both fat and muscle cells are abundantly present in these samples (Fig. 6e). We concluded from these data that XenofilteR can be applied to RNA sequencing data as well. Furthermore, we show that gene expression profiles can be generated of exclusively the murine compartment in PDX samples, despite the fact that murine sequence reads represented only a minor fraction of the total number of sequenced reads. Furthermore, based on the murine-specific gene expression profiles, we can identify the predominant cell types surrounding or infiltrating the PDX in the host.

## Discussion

High similarity between mouse and human genetics complicates the downstream analysis of both RNA and DNA profiles from tumor xenografts, including PDX models. The key problem is that a substantial percentage of sequence reads of mouse origin can be mapped with high quality to the human reference genome. These murine sequence reads map to conserved regions between mouse and human DNA, mainly exonic regions of the human genome, many of which correspond to cancer-related genes, e.g. *PTEN* and *BCL9*. Mutation analysis on PDX WES data revealed thousands of identified SNVs that were false positives, because they arose from mouse sequence reads. Importantly, we show that enrichment steps with human-specific baits also enrich for mouse DNA, likely due to the high DNA sequence similarity between mouse and human in the coding region of the genome, consistent with previous analyses [27–29].

Our in-depth analyses of sequence data from PDX samples demonstrate that the edit distance (the number of base pairs in a given mapped read discording with the reference genome) of a sequence read can distinguish the origin of the sequence reads. Sequence reads, aligned to a reference genome by most standard tools, provide the metrics to calculate the edit distance. On the basis of this premise, we developed XenofilteR, a method to classify sequence reads based on edit distances of sequence reads (Additional file 6: Figure S3). Because the edit distance can be calculated from mapped sequence files from the most frequently used sequence mappers, XenofilteR is a highly versatile method that can easily be implemented in current sequence analysis pipelines. Mapping of the sequence data can be done with the trusted and validated mapper of choice (e.g. BWA [23] or Tophat2 [24]), since XenofilteR requires only a CIGAR string and NM-tag, standard values present in the BAM-format [31]. XenofilteR has been implemented in the open-source programming language R. The R-package provides access to the method for investigators without programming knowledge by easy installation [26], extensive documentation and example data. In addition, we provide a Perl implementation to facilitate alternative integration for more advanced programmers of the XenofilteR algorithm in existing pipelines. We have used an earlier version of this algorithm already for the analysis of PDX models by targeted sequencing, both WES and a small cancer gene panel, and it proved to be an important tool for the identification of mutations causing resistance to targeted therapy [14, 15].

The validation results of XenofilteR using in silico mixed samples and PDX samples revealed accurate filtering, allowing for reliable VAF and accurate mutation calling. For example, all mutations detected in patient sample M032 (*n* = 419) were also detected after mixing of the sample with mouse reads and subsequent filtering with XenofilteR. XenofilteR appeared to filter more sensitively in multiple genomic regions than the Xenome algorithm, identifying more mutations with a more accurate VAF. Compared to Bamcmp, XenofilteR filters more thorough, thereby removing more sequence reads of mouse origin without losing somatic mutations. Although no real mutations were missed in the in silico validation, 15 false-positive mutations were identified after filtering with XenofilteR. Interestingly, most of the sequence reads underlying the false positive SNVs and erroneously classified as ‘human’ were not mapped to the mouse reference genome, despite being of mouse origin. The importance of a good reference genome is also reflected by the in silico admixed WGS sample where classification of the mouse strain C57BL/6NJ yielded fewer mouse reads after filtering compared to the BALB/cJ strain, this because the reference genome is based on the C57BL/6NJ. This exemplifies the importance of good and accurate reference genomes for any filtering to work and suggests a reference genome based on the mouse host will yield the best results.

In addition to removing mouse reads from PDX data, we demonstrate here the feasibility of analyzing the mouse stromal environment associated with (PDX) human tumor xenografts. We show that expression profiles typical for two major mouse cell types (fat cells and muscle cells) can be recovered from the PDX RNA sequence data when isolating for the mouse sequence reads. The high variability in the number of mouse-derived sequence reads per sample will pose a challenge when comparing the gene expression between samples. However, the possibility to analyze mouse gene expression in tumor xenografts will likely help understand interactions between tumor cells and stromal cells.

## Conclusions

In conclusion, we present here the XenofilteR algorithm as a solution for the problem of intermingled murine host and human cells in tumor xenografts. XenofilteR can be applied to both DNA and RNA sequencing and uses the edit distance, providing a straightforward and fast implementation that outperforms currently available methods.

## Methods

### PDX samples

The collection and use of human tissue was approved by the Medical Ethical Review Board of the Antoni van Leeuwenhoek. Animal experiments were approved by the animal experimental committee of the institute and performed according to Dutch law. PDX tumor fragments of ~ 5 mm^3^ were used for subcutaneous transplantation into NOD.Cg-*Prkdcscid Il2rgtm1Wjl/* SzJ (NSG) mice, which was performed under anesthesia. Before reaching the maximum allowed tumor size, mice were sacrificed, tumors were removed, and tumor pieces were (1) fixed in formalin and embedded in paraffin; (2) snap-frozen and stored at − 80 C for further analyses; (3) cryopreserved in 10% fetal calf serum (FCS) in DMSO and stored at − 80 C for additional passages; or (4) re-transplanted into a new set of NSG mice [14].

### Next generation sequencing

PDX tumor material was extracted as previously described [14]. Briefly, melanoma PDX samples from 6 patients and 6 matched blood samples were analyzed with WES [14]; 10 melanoma PDX samples were analyzed with targeted sequencing [14]; 6 melanoma PDX samples and matching patient tumor samples were analyzed with RNAseq^13^ and 3 breast cancer PDX samples with matching blood normal as well as matching patient tumor were also analyzed with WES [14].

Whole exome sequencing (WES) was performed as described in Kemper et al. [14]. Exome enrichment was performed using the Agilent SureSelect Human Exon Kit 50 Mb capture set (Agilent, G3362). Paired-end 75 reads of targeted-enrichment libraries were sequenced on the HiSeq 2000. Reads were mapped to the Ensembl human reference (hg19) by bwa 0–7.5 with default settings [23]. BAM files were processed using Picard [1.101] (http://picard.sourceforge.net), SAMtools [0.1.18 and 0.1.19] [31] and the Genome Analysis ToolKit (GATK) release 2.7–4. The sequencing data has been made available through the European Genome-phenome Archive (EGA; http://www.ebi.ac.uk/ega/home; accession number EGAS00001000415 and EGAD00001000869). WES of mouse DNA, enriched using a human enrichment kit (SureSelect Human Exon Kit 50 Mb capture set, Agilent, G3362) was sequenced as described. The sequencing data has been made available through the European Genome-phenome Archive (ENA; http://www.ebi.ac.uk/ega/home; accession number PRJEB23702).

### Merged mouse with human sequence data

For validation of XenofilteR and comparison of XenofilteR with other tools we generated WGS and WES files with artificially mixed mouse and human reads.

WGS data was generated by merging data from WGS from mouse (BALB_cJ And C57 downloaded from: www.sanger.ac.uk/resources/mouse/genomes/) [27, 28] and WGS from the 1000Genomes project (NA12878, source: www.internationalgenome.org/data-portal/) [29]. Paired-end sequencing with 100 bp was available for mouse WGS as well as for human WGS. To ensure all reads were mappable, only mapped read pairs were extracted from bam files and converted to fastq using Picard SamToFastq. Read pairs containing adapters or uncertain base calls (N) in either read were filtered.

Using these filtered reads, we generated single-end and paired-end fastq files with 50 bp, 80 bp or 100 bp reads. Mouse and human reads were randomly chosen and mixed in 6 different ratios with 100%, 94%, 88%, 75%, 50% and 0% human reads. All fastq files contained 70 Million reads (SE) or read pairs (PE). The resulting 72 sets were mapped in parallel to GRCh38 and mm10 before analysis with XenofilteR, or directly provided to the Xenome pipeline.

The identical strategy was used for the WES data using sequence reads of melanoma tumor (M032) [14] sequence reads from mouse DNA, enriched with a human enrichment kit, in a 4:1 human/mouse ratio.

### RNA sequencing

Three sets of RNA sequencing data were used: 3 mouse strains (NOD/ShiLtJ, BALB/cJ, C57BL/6NJ) [27, 28], 7 PDX samples for which matched PDX samples were available [14], and 95 PDX samples without matched patient samples. The mouse strains downloaded from The PDX samples with matched patient samples were processed as previously described [14].

For the second set, all 95 PDX samples were uniquely barcoded and pooled into a single stranded library and sequenced. An average of 56 million unique read pairs were sequenced per sample in a range between 33 and 78 million. All samples were mapped with Tophat2 v2.1.0 with the following parameters: --library-type fr-firststrand -g 1 -p 8 -G ENSEMBL_Annotation_v82.gtf. All samples were mapped to human reference GRCh38 (ENSEMBL v82) and mouse reference genome GRCm38 (ENSEMBL v82). The resulting alignments to human and mouse references were provided to XenofilteR (version 1.4, with default settings), to select for reads of mouse origin. Bam files were name sorted with picard tools followed by counting reads with HTseq-count (HTSeq-0.6.1p1) with settings: -m intersection-nonempty -a 10 -i gene_id -s reverse -f bam. Count data generated with HTseq-count was analyzed with DESeq2 [32].

### Mutation calling

Variants were called by GATK 2.7–4 using the ‘UnifiedGenotyper’ with default settings, except for “-minIndelFrac”, which was set to 10%. Annotation of the vcf files was performed with annovar (http://annovar.openbioinformatics.org). All variants detected in the germ-line (blood) samples with a Variant Allele Frequency (VAF) over 2% were excluded from further analysis. Variants were further filtered: minimum VAF of 0.1 in at least one of the samples; a minimum of 20× coverage in at least one of the samples; variant positions, listed as a single nucleotide polymorphism (SNP) in the 1000 Genome project, were excluded, except when also present in COSMIC [33]; Variant position were kept only if annotated as exonic by RefSeq (Release 45) and only if the change was non-synonymous.

### XenofilteR

All data shown in this paper were analyzed with XenofilteR version 1.4 as available through Github, http://github.com/PeeperLab/XenofilteR/releases/tree/V1.4. All samples described here were processed with the default settings.

### Xenome

Xenome version 1.0 [19] was downloaded from the following website: https://github.com/data61/gossamer and run using default settings. Fastq output files were altered to comply with bwa and allow mapping; by prepending an ‘@’ to each read name and a ‘+’ to each separator line.

### ‘Strict filtering’

Filtering of xenograft samples using the ‘strict’ filtering method, removal of each read that maps to the mouse reference, was performed using a custom version of Xenofilter based on version V1.4: https://github.com/PeeperLab/XenofilteR/tree/Strict_filtering. Identical to the normal XenofilteR version, classification of reads was based on primary alignments only identified by the read name. For paired-end sequencing if either the forward of reverse reads mapped to the mouse reference genome, the read pair was classified as ‘mouse’.

### BBsplit

BBsplit is available in the BBmap software (Version 38.12) and was downloaded from: https://sourceforge.net/projects/bbmap/ [22]. BBSplit was run using default settings.

### IHC and sanger sequencing

PDX pieces were fixed in formalin and embedded in paraffin. Slides were stained by our in-house Animal Pathology Facility for H&E as previously described [14].

Sanger sequencing to confirm the SNPs was performed as follows: the region of interest was amplified by conventional PCR, which then was sequenced by the forward primer or a specific sequencing primer. The following human specific primers were used:

*MEN1-F (rs7121): TCCCTCACCTGTCCCTCAAA;*

*MEN1-R (rs7121): CTGATCTGTGCCTCCCTTC;*

*GNAS-F (rs2071313): GTTCCCTGACCGCTTTGCTA;*

*GNAS-R (rs2071313): CACAAGTCGGGGTGTAGCTT.*

## Availability and requirements

Project name: XenofilteR.

Project home page: https://github.com/PeeperLab/XenofilteR

Operating system(s): Platform independent

Programming language: R

Other requirements: -

License: GNU General Public License v3.0

Any restrictions to use by non-academics: see Licence.

## Additional files

Additional file 1: **Table S1.** WGS of mouse strains mapped to human. Number of sequence reads from 3 mouse strains mapped to human genes (Tab. 1) and exons (Tab. 2). (XLSX 11940 kb)

Additional file 2: **Table S2.** Pathology estimate and SNVs of eight PDX samples. Percentage of mouse stromal cells, detected SNV and overlap with mouse derived SNVs of eight melanoma PDX samples. (XLSX 35 kb)

Additional file 3: **Table S3.** Number of sequence reads after filtering of the in silico dataset. Number and percentages of mouse and human reads left after filtering with strict filtering, bamcmp, BBsplit, Xenome and XenofilteR for all read lengths, paired and single end sequencing and percentages tested. (XLSX 36 kb)

Additional file 4: **Figure S1.** Performance of XenofilteR and Xenome on PDX samples. Mutation calling and read counts for each SNV on exome sequence data of a breast cancer PDX sample T250 (A) and T283 (B). The variant allele frequency (VAF) was plotted after filtering with XenofilteR (x-axis) and Xenome (y-axis). Plotted in black are mutations also detected in the patient sample, in green known SNPs and in red SNVs detected in the PDX only. C: Mutation calling on targeted sequencing of melanoma samples. In green all known SNPs are indicated, in black the remaining SNVs. (TIF 3452 kb)

Additional file 5: **Figure S2.** Validation of mutation calling after filtering with Xenofilter and Xenome. A: IGV image of SNP rs2071313, located in the gene *MEN1*, of sample M041.X1 and M046.X1 after filtering with Xenome and XenofilteR. B: Validation of the SNP rs2071313 (*MEN1*) by Sanger sequencing with human-specific primers. (TIF 3324 kb)

Additional file 6: **Figure S3.** Graphical abstract of Xenograft sequence analysis with XenofilteR. Sequence data obtained from xenograft samples contains sequence reads from mouse as well as sequence reads from human origin. XenofilteR separates these reads allowing further downstream analysis based on sequence reads of human origin only. (TIF 3293 kb)

## Abbreviations

FPKM: Fragments per kilobase of exon per million reads mapped
PDX: patient derived xenograft
SNP: single nucleotide polymorphism
SNV: single nucleotide variant
WES: whole exome sequencing
WGS: whole genome sequencing
