# Supplementary figures and images for: XenofilteR: computational deconvolution of mouse and human reads in tumor xenograft sequence data
Source: BMC Bioinformatics. 2018 Oct 4;19:366. doi: 10.1186/s12859-018-2353-5 (PMC6172735; doi:10.1186/s12859-018-2353-5)

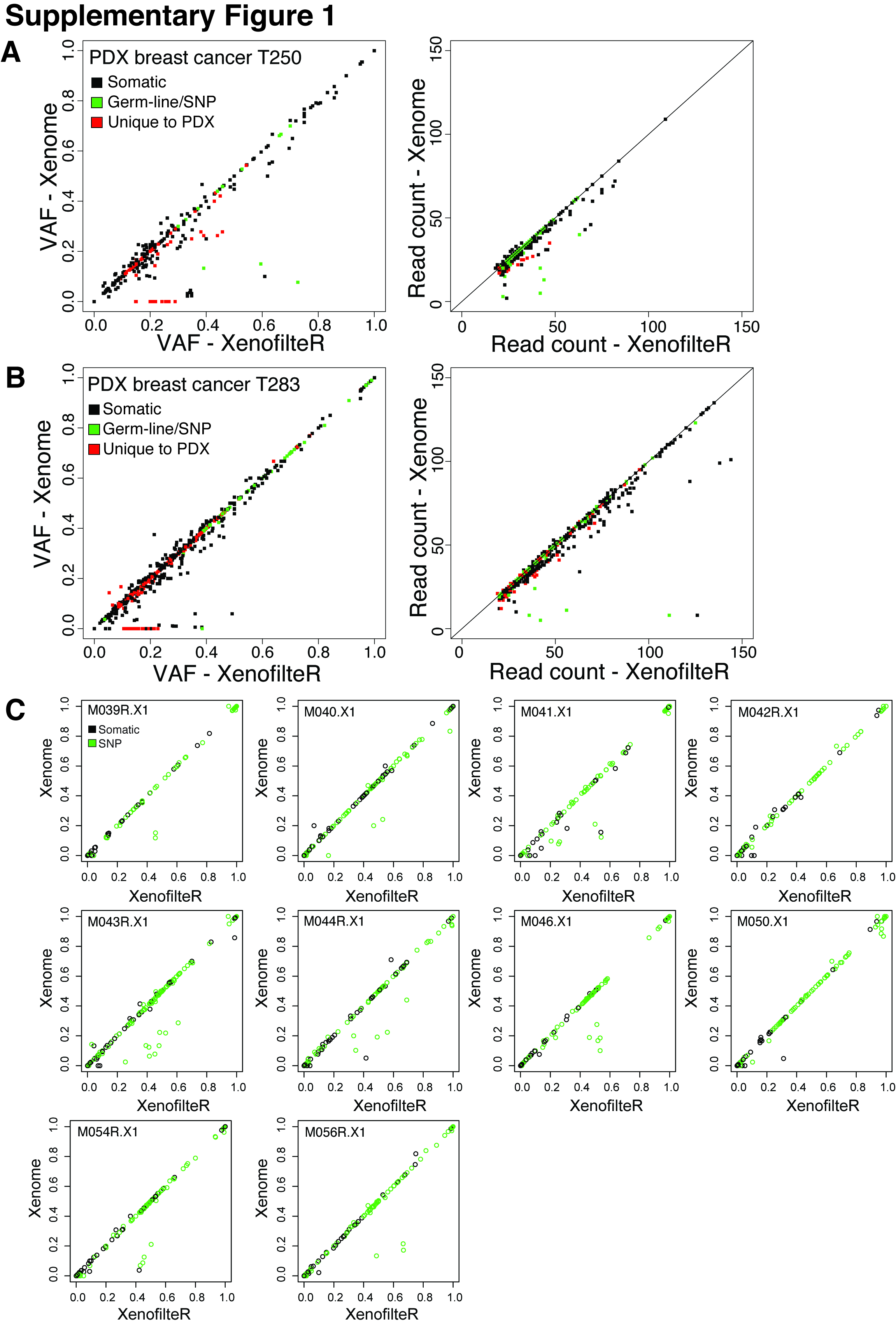

Supplement: Supplementary file 4 — Figure S1. Performance of XenofilteR and Xenome on PDX samples. Mutation calling and read counts for each SNV on exome sequence data of a breast cancer PDX sample T250 (A) and T283 (B). The variant allele frequency (VAF) was plotted after filtering with XenofilteR (x-axis) and Xenome (y-axis). Plotted in black are mutations also detected in the patient sample, in green known SNPs and in red SNVs detected in the PDX only. C: Mutation calling on targeted sequencing of melanoma samples. In green all known SNPs are indicated, in black the remaining SNVs. (TIF 3452 kb) [file 12859_2018_2353_MOESM4_ESM.tif]

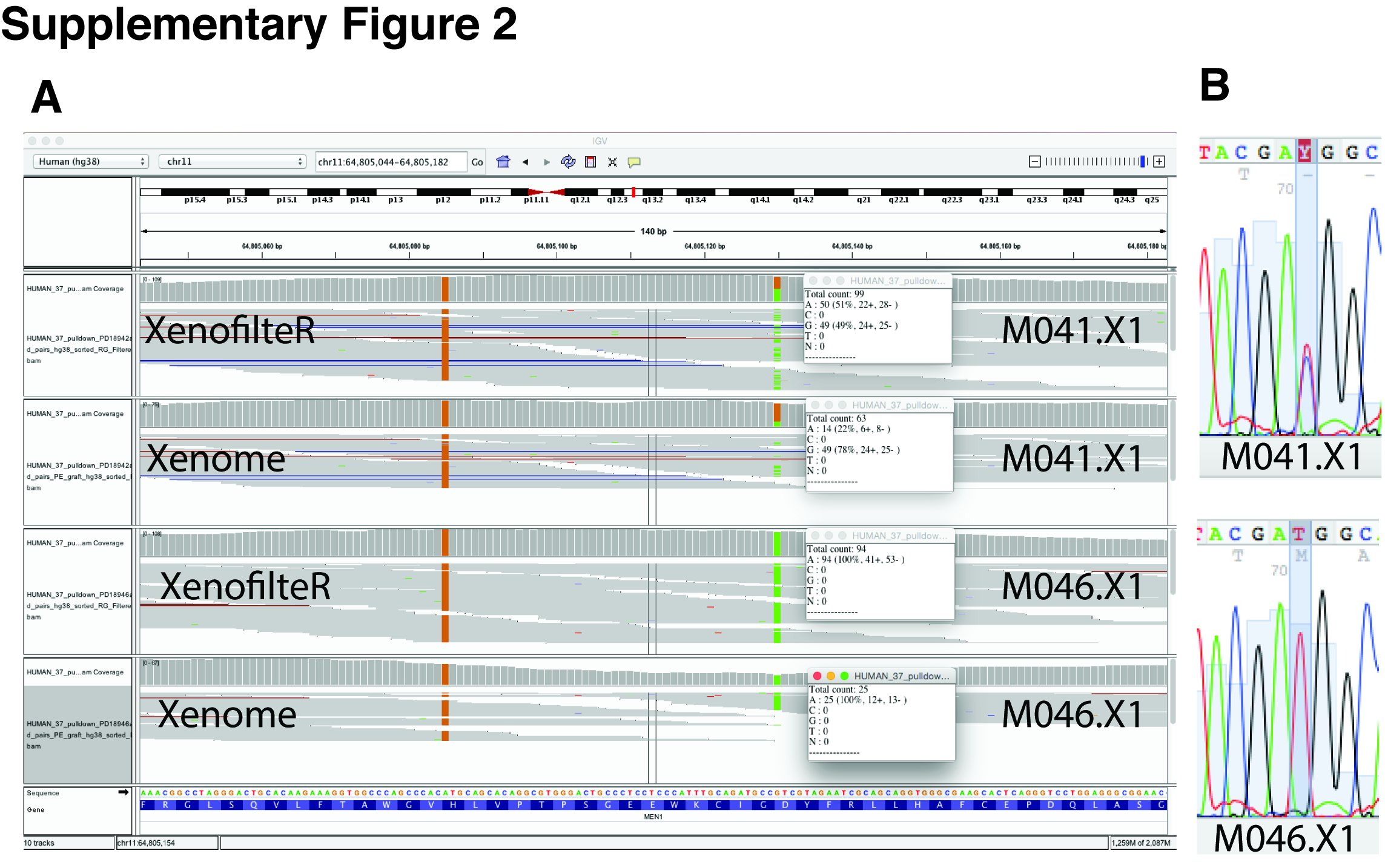

Supplement: Supplementary file 5 — Figure S2. Validation of mutation calling after filtering with Xenofilter and Xenome. A: IGV image of SNP rs2071313, located in the gene MEN1, of sample M041.X1 and M046.X1 after filtering with Xenome and XenofilteR. B: Validation of the SNP rs2071313 (MEN1) by Sanger sequencing with human-specific primers. (TIF 3324 kb) [file 12859_2018_2353_MOESM5_ESM.tif]

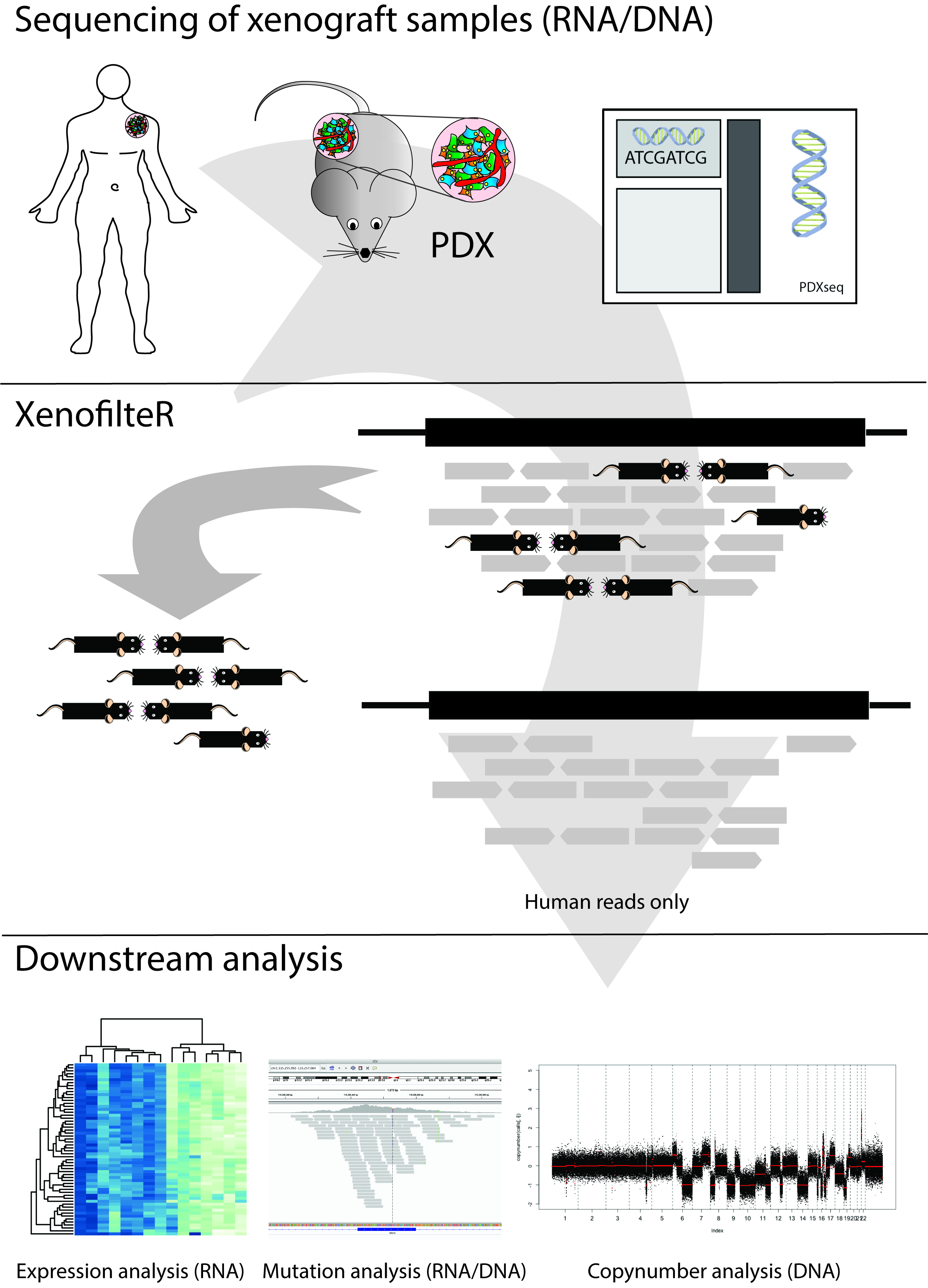

Supplement: Supplementary file 6 — Figure S3. Graphical abstract of Xenograft sequence analysis with XenofilteR. Sequence data obtained from xenograft samples contains sequence reads from mouse as well as sequence reads from human origin. XenofilteR separates these reads allowing further downstream analysis based on sequence reads of human origin only. (TIF 3293 kb) [file 12859_2018_2353_MOESM6_ESM.tif]
